# Evaluation des complications cardiaques chez les hémodialysés chroniques de Dakar

**DOI:** 10.11604/pamj.2016.23.43.7227

**Published:** 2016-02-15

**Authors:** Cissé Mouhamadou Moustapha, Lemrabott Ahmed Tall, Faye Maria, Fall Khodia, Faye Moustapha, Ka El Hadji Fary, Niang Abdou, Diouf Boucar

**Affiliations:** 1 Service de Néphrologie du CHU A Le Dantec, Dakar, Sénégal

**Keywords:** Hémodialyse, Insuffisance rénale chronique, Complications cardiovasculaires, Sénégal, Hemodialysis, chronic renal failure, cardiovascular complications, Senegal

## Abstract

**Introduction:**

L’évaluation cardiovasculaire est essentielle en hémodialyse périodique car les affections cardiovasculaires sont la première cause de mortalité chez les hémodialysés chroniques. Nous avons conduit cette étude afin de déterminer la prévalence et le type des différentes complications cardiovasculaires et d'identifier les principaux facteurs de risque cardiovasculaire.

**Méthodes:**

Il s'agissait d'une étude rétrospective de 4 ans portant sur les dossiers de patients traités au moins 6 mois en hémodialyse et ayant des explorations cardio-vasculaires comportant une radiographie du thorax de face, un électrocardiogramme et une échographie cardiaque. Les données épidémiologiques, cliniques, paracliniques, les aspects évolutifs des complications cardiaques ont été recueillies pour chaque patient retenu.

**Résultats:**

Trente huit dossiers de patients ont été inclus dans cette étude. L’âge moyen était de 52 ans ± 12,85 et le sex-ratio H/F de 1,53. Les complications cardiovasculaires étaient dominées par l'hypertrophie ventriculaire gauche (71,05%), la maladie coronaire (34,21%), l'insuffisance cardiaque congestive (18,42%), Calcifications vasculaires (5,78%), les troubles du rythme (7.89%), les fuites valvulaires mitrales (44,73%), tricuspidiennes (42,10%) et les accidents vasculaires cérébraux hémorragiques (5,26%). L'incidence moyenne de l'HVG était de 81% chez les patients hypertendus. Au cours de cette étude, 27 patients avaient poursuivi l'hémodialyse et 11 étaient décédés dont 6 de causes cardiovasculaires (54,55%).

**Conclusion:**

L'hémodialyse est une technique d’épuration qui expose les patients à de multiples complications cardiovasculaires.

## Abstract (en)

**Introduction:**

Hemodialysis is the first extrarenal treatment method that allowed supporting patients in terminal chronic failure in Senegal since 1997. 25 years later, we conducted this study to determine the type and the prevalence of different cardiovascular complications and identify the main cardiovascular risk factors.

**Methods:**

It is a retrospective study of 4 years. 38 patients treated at least 6 months in hemodialysis and cardiovascular explorations with a front chest x-ray, electrocardiogram and cardiac ultrasound. All patients who have not started hemodialysis, treated less than 6 months in hemodialysis, treaties in peritoneal dialysis or having raised cardiovascular explorations were excluded. For each selected patient, we collected data epidemiological, clinical, paraclinical and evolutionary aspects of cardiovascular complications.

**Results:**

38 patients were included in this study. The average age was 52 years ± 12.85 and the sex ratio H/F of 1.53. Initial nephropathy was dominated by the néphroangiosclérose followed by diabetic nephropathy. Clinically the signs of appeal are marked by the effort dyspnea palpitations, chest pain and physically by the HTA, anemia. Cardiovascular complications were dominated by hypertrophy (LVH) left ventricular, rhythm type of arrhythmia disorders valvular leakage (mitral and tricuspid) and cerebral vascular accident (stroke). The average impact of LVH according the HTA is 81%, by sex of 78.26% for men and 60% for women. At the end of the study, 27 patients were pursuing hemodialysis and 11 had died 6 (54%) of cardiovascular cause.

**Conclusion:**

Hemodialysis is a common purification technique in Senegal and its complications remain especially dominated by abuses cardiovascular.

## Introduction

La prise en charge des patients hémodialysés s'est nettement améliorée mais avec une augmentation des complications cardiovasculaires qui constituent la principale cause de morbidité et de mortalité [1]. Le taux de mortalité dû aux maladies cardiovasculaires chez les patients dialysés est de 10 à 30 fois plus élevé que dans la population générale [2]. Toutes les tuniques cardiaques peuvent être intéressées par ces complications [1, 3, 4]. Dans le but de mieux caractériser ces atteintes, nous avons conduit cette étude afin de déterminer la prévalence des différentes complications cardiaques.

## Méthodes

Il s'agissait d'une étude rétrospective, transversale. Elle s'est déroulée sur une période de 4 ans allant de 2005 à 2009 au CHU Aristide le Dantec de Dakar. Tous les dossiers des patients en hémodialyse périodique pendant au moins 6 mois et ayant un bilan cardiologique ont été inclus. Ces dossiers contenaient l’âge, le sexe, le tabagisme, la néphropathie initiale, le diabète, la pression artérielle pré dialytique, la prise de poids inter dialytique (PPID) et la dose de dialyse ou Kt/V. Ce dernier permet d'apprécier l'efficacité de la dialyse et une valeur supérieure ou égale à 1,3 a été retenue comme normale. L'hypertrophie ventriculaire gauche était définie par une masse ventriculaire gauche indexée à la surface corporelle supérieure à 131g/m^2^ chez l'homme et 100g/m^2^ chez la femme. L'hypertension artérielle pulmonaire était définie par une pression artérielle pulmonaire systolique supérieure à 35 mm Hg. Tous les patients ont bénéficié d'une radiographie du thorax de face, d'un électrocardiogramme et d'une échographie cardiaque. Les dossiers des patients incomplets ont été exclus de l’étude. Les données ont été analysées à l'aide du logiciel SPSS version 16.0, 2007. Les variables quantitatives ont été exprimées en moyennes ± écart type et les variables qualitatives en effectif et en pourcentage. Le test t de Student a été utilisé pour la comparaison des variables quantitatives et le test Chi 2 ou celui de Fisher exact pour la comparaison des variables qualitatives. L'analyse multivariée a fait appel à la régression logistique multiple. Une valeur p < 0,05 a été considérée comme significative.

## Résultats

**Figure 1:**
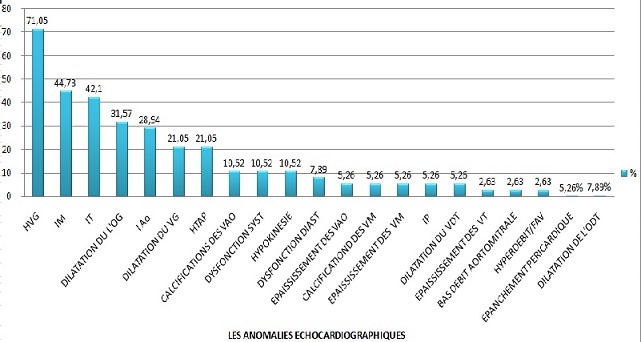
Anomalies écho cardiographiques

Au total, 38 dossiers de patients hémodialysés étaient retenus pour l’étude. L’âge moyen était de 52 ans ± 12,85 et le sex-ratio H/F de 1,53. La néphropathie initiale était dominée par la néphroangiosclérose et la néphropathie diabétique respectivement chez 20 et 4 patients (Tableau 1). Les signes physiques sont représentés respectivement sur le Tableau 1. Quant aux signes radiologiques, l'index cardiothoracique moyen (ICT) était de 0,53±0,06 (extrêmes: 0,42-0,76). Vingt-quatre patients avaient une cardiomégalie aux dépends du ventricule gauche (63,16%), tandis que 14 (36,84%) avaient un ICT inférieur à 0,5, et un seul patient avait un arc moyen gauche convexe évocateur d'une hypertension artérielle pulmonaire (HTAP). Les anomalies électriques portaient respectivement sur: l'hypertrophie ventriculaire gauche, l'hypertrophie auriculaire gauche et les troubles de la repolarisation respectivement chez 27 (71,05%), 10 (26,32%) et 13 (34,21%) patients (Tableau 2). Le Tableau 3 résume les moyennes des différents paramètres biologiques retenus pour l’étude. Les anomalies écho cardiographiques sont représentées sur la Figure 1. Sur le total des patients, la durée moyenne en hémodialyse était de 35,39 ± 13,71 mois (extrêmes: 9-48 mois). Huit patients étaient hémodialysés 12 heures par semaine, contre 30 qui bénéficiaient de 10 heures de séance par semaine. Vingt neuf patients (76%) avaient une prise de poids interdialytique (PPID) entre 3 et 4 Kg tandis que 9 (24%) avaient une PPID de plus de 5 Kg. L'abord vasculaire pour l'hémodialyse était une fistule artérioveineuse (FAV) chez tous les patients (100%). Le siège était distal chez 30 patients (78,95%) et proximal chez 7 (21,05%) dont 4 avaient une complication à type d'hyperdébit. Le KT/V a été mesurée chez 19 patients. La moyenne était de 1,85 + /-0,44(extrêmes: 1,19-2,98). Dix-huit patients avaient un taux normal et 1 avait un taux bas. En analyse multivariée, l’âge, le sexe, la prise de poids interdialytique, le siège de la fistule artério-veineuse, l'hyperparathyroïdie, l'hypoalbuminémie, le nombre d'heures de dialyse par semaine et le KT/V n’étaient pas associés à l'HVG. En revanche, l'HTA et l'anémie ont été identifiés comme déterminants majeurs de la survenue de l'HVG chez les patients hémodialysés (différence significative avec respectivement p < 0,036 et 0,019). Il n'existait pas de différence statistiquement significative entre la survenue des troubles de la repolarisation et l'anémie (p= 0,06), la dyslipidémie (p = 0,5), l'HTA (p = 0,17), le diabète (p= 0,64) et le taux de CRP (p = 0,4). Concernant les calcifications valvulaires cardiaques, il n'y a pas de facteurs qui ont été décelés dans leur survenue notamment l’âge (p= 0,61) et l'ancienneté en hémodialyse (p= 0,61). Ceci peut s'expliquer aussi par la faible taille de notre échantillon et les écarts d’âge peu importants. Onze patients étaient décédés (29%) dont la cause de décès était dans 6 cas liée à une atteinte cardiovasculaire (54,55%). Les décès étaient liés à un infarctus du myocarde (2cas: 18,18%) et à un accident vasculaire cérébral (2 cas: 18,18%). Dans 2 cas (18,18%), la cause du décès était indéterminée.

**Tableau 1:** Néphropathies initiales et signes physiques

**Néphropathies initiales**	
Glomérulonéphrite chronique	52,63%
Néphropathie indéterminée	10,53%
Néphropathie bilharzienne	2,63%
Néphropathie diabétique	10,53%
Néphroangiosclérose	7,89%
Néphropathie tubulo-interstitielle chronique	5,26%
Polykystose rénale autosomique dominante	7,89%
Cancer du rein	2,63%
**Signes physiques**	
HTA	27 cas
Anémie clinique	29 cas
Arythmie cardiaque	3 cas
Frottement péricardique	1 cas
Insuffisance cardiaque	10 cas

**Tableau 2:** Anomalies électriques

Tachycardie sinusale	4 cas
Bradycardie sinusale	2 cas
Hypertrophie ventriculaire gauche	27 cas
Hypertrophie auriculaire gauche	10cas
Hypertrophie biauriculaire	1 cas
Extrasystole ventriculaire monomorphes isolées	2 cas
Flutter atrial	1 cas
Troubles de la conduction	6 cas
Bloc de branche droit complet	1cas
Bloc de branche gauche complet	1cas
Hémibloc antérieur	3cas
Bloc auriculoventriculaire du 1^er^ degré	1 cas

**Tableau 3:** Différents paramètres biologiques étudiés

Biologie	Nombre	moyenne	Ecart type	Min	Max
**Taux d'hémoglobine**	38	8,89	1,81	6,2	13,1
**Ferritinémie**	34	760,66	1225,36	16	7113
**Protides**	31	75,75	5,41	67	91
**Albumine**	31	40,50	6,23	25	51
**Calcium**	37	92,18	10,52	63	119
**Phosphore**	37	51,78	21,67	23	113
**PTHi**	30	307,21	281,44	14	994

## Discussion

Dans notre étude, le taux moyen de l'HVG (71,05%). C'est le cas de Bah A. O. et al. en 2006 en République de Guinée (72,95%) [3] et tout récemment de A. Aldlouni et al. en 2011 au Maroc (87%) [1]. Ce taux élevé d'HVG s'explique par la fréquence de l'anémie, de l'HTA et de la prise de poids excessive chez ces patients. Une attention particulière sera accordée au suivi de l'anémie et de l'HTA d'autant plus qu'ils ont été identifiés comme déterminants majeurs de la survenue de l'HVG. Ce fait a été aussi rapporté par Eba A., Aghrabat M.S. en 2006 en Mauritanie [4]. Il en est de même de la FAV proximale à haut débit qui est reconnue comme un facteur favorisant l'HVG [5, 6]. Statistiquement ceci conforte les résultats de notre étude. Parmi nos 8 patients qui avaient une FAV proximale, 4 avaient un hyperdébit. Au cours du suivi, la proportion des troubles de la repolarisation étaient retrouvés chez 13 patients soit 34,21% avec un âge moyen de 50,4 ans. Ce résultat est similaire à ceux retrouvés par SABOURI [7] où l’âge moyen était de 55,7 ans. Dans la littérature comme le rapporte l’étude de B. CHARRA [8], la prévalence élevée de l'hypertrophie ventriculaire gauche, de l'hypertension artérielle et du diabète chez les hémodialysés est à l'origine d'une maladie coronaire souvent silencieuse et associée à des troubles de la repolarisation non spécifiques sur l'ECG de repos. Ceci conforte notre étude où nous avons établi une corrélation significative entre l'HVG et la survenue des troubles de la repolarisation. Cependant, il n'y avait pas de corrélation entre les troubles de la repolarisation et le diabète ainsi qu'avec l'HTA avec une différence non significative dans les 2 cas ce qui s'explique entre autres par la faible taille de l’échantillon. Ce constat va à l'encontre des résultats d'autres études qui ont recherché les facteurs de risque cardio-vasculaires chez l'hémodialysé. Notamment TAKEDA et Al. [9] au Japon, qui ont démontré que le risque de survenue de nouveaux évènements cardio-vasculaires était fortement lié à l'HTA (p< 0,0005).

La prévalence des troubles de la conduction dans notre série était de 15,79%. Le même résultat a été noté par GERGAUD [10] avec 15,3% des troubles de la conduction chez les hémodialysés. Chez 71,05%, des patients l’échographie cardiaque avait mis en évidence une HVG, qui pourrait être à l'origine de ces troubles de la conduction. Cette théorie est réconfortée par JUNGERS P. [11] qui avait montré dans sa série que l'HVG et les calcifications de la jonction auriculo-ventriculaire étaient les principaux facteurs responsables des troubles de la conduction. Trois patients (7,89%) avaient des troubles du rythme. Ils avaient à la fois une HVG, une prise excessive du poids interdialytique et des troubles de la repolarisation. Des résultats similaires ont été retrouvés par JUNGERS P. [9] et EBA A [12] dans respectivement 12% et 3,5%. Nous avons retrouvé l'HTAP chez 8 patients (21,05%). JUNGERS [11] avait retrouvé dans sa série une HTAP dans 20%. L'HVG, la FAV à haut débit, les troubles métaboliques et hormonaux associés à l'IRCT et le tabagisme ont été évoqués comme les facteurs étiologiques possibles d'HTAP chez les hémodialysés. Dans notre série, les calcifications des valves mitrales ont été retrouvées dans 5,26% et aortiques dans 10,52% des cas. Ce qui est similaire aux résultats retrouvés en Mauritanie où les calcifications des valves mitrales étaient mises en évidence respectivement dans 12% et 3% des cas sans calcifications des valves aortiques [4]. Une corrélation a été retrouvée entre l’âge, la durée d'hémodialyse et la présence des calcifications valvulaires dans la littérature [13]. Aucune corrélation n'a été retrouvée entre les calcifications valvulaires chez nos patients et l'ancienneté en dialyse. Ce pendant dans une étude japonaise chez les hémodialysés (1290) patients suivis pendant 10ans (2000-2010) publiée en Févier 2013, il a été rapporté que les patients atteints de calcification de la valve étaient plus âgés et CRP sérique et de l'hormone parathyroïdienne intacte étaient plus élevés; inversement, l'indice de masse corporelle et le taux d'albumine et de la créatinine sériques étaient plus faibles chez les patients présentant une calcification de la valve que chez ceux sans [2]. Ceci pourrait être expliqué par la durée courte de nos patients en dialyse (moyenne inférieure à 2 ans) par rapport à celle décrite dans la littérature. Les décès dans notre étude étaient liés à une atteinte cardiovasculaire dans 54,55%. Les IDM et les AVC venaient au premier plan avec chacun un pourcentage de 18,18% des cas. En Afrique, EBA en Mauritanie avait retrouvé que 62% de décès étaient d'origine cardiovasculaire chez les hémodialysés [3] de même qu'aux USA et au Japon où les causes cardiovasculaires de décès chez les hémodialysés étaient notées respectivement dans 52% et 58% des cas [12, 14].

## Conclusion

Les complications cardiaques sont fréquentes chez les patients sénégalais hémodialysés à Dakar. Leur prévention passe par un bon contrôle des principaux facteurs de risque à savoir l'HTA et l'anémie. La réalisation régulière d’échocardiographies permettrait leur dépistage précoce afin de pouvoir proposer un traitement optimal.

### Etat des connaissance sur le sujet

- Les complications cardiovasculaires constituent la première cause de morbimortalité chez les patients hémodialysés chroniques.
- Elles sont dominées par l'hypertrophie ventriculaire gauche, mais peuvent affecter toutes les tuniques cardiaques et vasculaires.

### Contribution de notre étude a la connaissance

- Notre étude est venue confirmée encore que l'hypertrophie ventriculaire gauche est la principale complication cardiaque chez les hémodialysés chroniques.
- Ce travail montre en plus que les calcifications cardiaques ne sont pas corrélées à l'ancienneté en hémodialyse.
